# Peer victimisation and its association with psychological and somatic health problems among adolescents in northern Russia

**DOI:** 10.1186/1753-2000-7-15

**Published:** 2013-05-14

**Authors:** Andrew Stickley, Ai Koyanagi, Roman Koposov, Martin McKee, Bayard Roberts, Vladislav Ruchkin

**Affiliations:** 1 Stockholm Centre on Health of Societies in Transition (Scohost), Södertörn University, Huddinge, Sweden; 2 Centre for Child and Adolescent Mental Health and Child Welfare, Institute of Clinical Medicine, University of Tromsø, Tromsø, Norway; 3 European Centre on Health of Societies in Transition, London School of Hygiene and Tropical Medicine, Keppel Street, London, UK; 4 Department of Child and Adolescent Psychiatry, Institute of Neuroscience, Uppsala University, Uppsala 75185, Sweden

## Abstract

**Background:**

A growing body of evidence from countries around the world suggests that school-based peer victimisation is associated with worse health outcomes among adolescents. So far, however, there has been little systematic research on this phenomenon in the countries of the former Soviet Union. The aim of this study was to examine the relation between peer victimisation at school and a range of different psychological and somatic health problems among Russian adolescents.

**Methods:**

This study used data from the Social and Health Assessment (SAHA) – a cross-sectional survey undertaken in Arkhangelsk, Russia in 2003. Information was collected from 2892 adolescents aged 12–17 about their experiences of school-based peer victimisation and on a variety of psychological and somatic health conditions. Logistic regression analysis was used to examine the association between victimisation and health.

**Results:**

Peer victimisation in school was commonplace: 22.1% of the students reported that they had experienced frequent victimisation in the current school year (girls – 17.6%; boys – 28.5%). There was a strong relationship between experiencing victimisation and reporting worse health among both boys and girls with more victimisation associated with an increased risk of experiencing worse health. Girls in the highest victimisation category had odds ratios ranging between 1.90 (problems with eyes) and 5.26 (aches/pains) for experiencing somatic complaints when compared to their non-victimised counterparts, while the corresponding figures for boys were 2.04 (headaches) and 4.36 (aches/pains). Girls and boys who had the highest victimisation scores were also 2.42 (girls) and 3.33 (boys) times more likely to report symptoms of anxiety, over 5 times more likely to suffer from posttraumatic stress and over 6 times more likely to experience depressive symptoms.

**Conclusion:**

Peer victimisation at school has a strong association with poor health outcomes among Russian adolescents. Effective school-based interventions are now urgently needed to counter the negative effects of victimisation on adolescents’ health in Russia.

## Background

In the past twenty years a large body of research has emerged highlighting the variety of negative consequences that can result from being a victim of peer bullying at school. Studies have shown that victimisation is associated with a range of negative health outcomes that include physical effects such as headache, stomach ache and dizziness [1] as well as psychological effects that can include anxiety and depression [2,3]. Victimisation has also been linked to an increased risk for self harm and suicidal behaviour [4]. It is possible that these negative effects may even stretch beyond childhood as frequent victimisation in school has also been associated with an increased risk of experiencing anxiety disorders in early adulthood [5].

The current study will examine the effects of peer victimisation at school on health outcomes among adolescents in Russia. Although the occasional and chronic bullying of adolescents by peers is commonplace throughout Europe [6], there is some evidence that rates of both bullying and victimisation are comparatively high in the former Soviet countries – including Russia [1,7]. As yet, however, there have been few studies that have specifically focused on the phenomenon of adolescent violence or peer victimisation in individual countries in the former Soviet Union. This is an important research gap, especially in Russia. Some evidence suggests that Russian adolescents may be subject to a variety of differing forms of peer victimisation including physical violence and abuse [8] and that this may be impacting on both their physical and mental health [1,8]. Peer victimisation might even be associated with the high suicide rates that have recently been reported among older adolescents in the country [9].

By exploring the association between victimisation and a number of different somatic and psychological health outcomes using a measure that encompasses various forms of victimisation, the current study will build on earlier research undertaken in the framework of the Health Behaviour in School-aged Children (HBSC) study in which Russia was included [1]. This is an essential task as it has been suggested that the issue of bullying is still being neglected in Russian schools [9]. In such circumstances determining the precise link between victimisation and health is important not only in terms of highlighting this phenomenon and its potentially deleterious effects on health more generally, but also when it comes to designing specific interventions that will be effective in countering bullying and its effects [10].

## Methods

### Study participants

We used data from the Russian Social and Health Assessment (SAHA). Ethical permission for this survey was obtained from the Northern State Medical University in Arkhangelsk and Yale University School of Medicine and it was carried out in accord with the principles laid out in the Declaration of Helsinki, 1975. A description of the survey’s methodology has been presented elsewhere [11]. In brief, the instrument was administered to a representative sample of sixth to tenth grade students in the public school system in the northern Russian city of Arkhangelsk in 2003. These students came from randomly selected classes that were within schools which were themselves randomly selected from the list of schools in each of the city’s four districts. The sampling was designed to achieve numbers proportionate to the number of students in each district. Both parents (for their children) and students themselves were informed of their right to refuse to participate in the study. Students completed the survey in their classrooms during a normal school day. Written informed consent was given by all participants. From the 3000 survey booklets that were distributed the final study sample consisted of 2892 adolescents (a 96.4% response rate), 42.4% of whom were boys.

### Measures

The Social and Health Assessment (SAHA) instrument, which has been used previously in a number of international studies, included both new scales developed specifically for this survey and scales used previously with similar populations [12]. The peer victimisation scale was an adapted version of the Multidimensional Peer Victimisation Scale [13]. This shortened version contained 9 questions on experiencing forms of physical victimisation, social manipulation, verbal victimisation, attacks on property and an additional item to the original – ‘standing too close or touching’ in school (see Additional file 1). Students reported on the frequency of peer victimisation they had experienced in the current school year [scored as 0 (not at all) 1 (once) 2 (2–3 times) 3 (4 or more times)], with the total combined score ranging from 0 to 27. This measure was used in two ways in this study. First, since bullying is usually understood as a repetitive behaviour [14], when calculating the prevalence of victimisation, we followed earlier researchers [15] by using more than one instance of victimisation. Specifically, we defined ‘occasional’ victimisation in terms of reporting at least 2–3 instances of victimisation on any one of the 9 questions in the current school year. Those students who reported 4 or more instances of victimisation on any one of the nine questions were categorised as experiencing ‘frequent’ victimisation. Second, to examine the relationship between victimisation and health we used the full scale of scores ranging from 0 to 27. To determine whether a greater degree of victimisation had a more detrimental impact on health this scale was broken down into 6 categories with the cut-off score for the highest category (i.e. 11–27) being chosen on the basis that it provided a sufficient number of cases to allow statistical analyses to be undertaken for both boys and girls. The victimisation scale had a high degree of internal consistency (Cronbach’s α=0.84).

In terms of their physical well-being students were asked if they had experienced any of the following eight somatic symptoms in the past 30 days – headaches, stomach ache, aches/pains, nausea, feeling sick (unwell), problems with eyes, rashes/skin problems, and vomiting. The response options to this question were, ‘not true’, ‘somewhat true’ and ‘certainly true’. In the statistical analysis those students who responded that it was either certainly true or somewhat true were categorised as having experienced the symptom. Information was also collected on three aspects of psychological ill health. The past 30-day experience of depressive symptoms was examined using an adapted and shortened 10-item version of the Centre for Epidemiological Studies-Depression Scale (CES-D) [16]. Adolescents reported on their feelings and behaviour on the same 3-point response category scale ranging from ‘not true’ (scored 0) to ‘certainly true’ (scored 2). The total score ran from 0–20 with a higher score indicating the presence of more depressive symptoms. Modified versions of the CES-D have previously demonstrated excellent psychometric properties with adolescent populations [12], while there was a high degree of internal consistency in this study (Cronbach’s α=0.82). Anxiety symptoms were measured using a 12-item scale specifically created for the SAHA survey that combined items from three scales commonly used to assess anxiety in adolescents and children. Using the same response options and scoring system employed for depressive symptoms, a scale was created that ran from 0–24 with higher scores indicating more anxiety. We used the top quintile of scores as the cut-off point for both symptoms of depression and anxiety in the statistical analyses. Similar to the version used with American adolescents [12], in the current study, the scale demonstrated a high level of internal consistency (Cronbach’s α=0.86). Finally, the Child Post-Traumatic Stress-Reaction Index (CPTS-RI) was used to assess symptoms of posttraumatic stress occurring in the past 30 days. This scale which has been widely used in earlier research consisted of 20 items scored between 0 and 4 that gave a cumulative score ranging from 0–80 (Cronbach’s α=0.86). The cut-off score of 25 and above, used in the current study is commonly used to signify the presence of at least a moderate degree of posttraumatic stress [12].

### Statistical analysis

The analysis was restricted to those adolescents aged 13–17 years old as the number of individuals outside this age range was small (24 cases). The prevalence of victimisation and the various health conditions are presented in percentages with 95% confidence intervals. Logistic regression analysis was used to assess the relation between victimisation and different health problems while controlling for the potential effects of age, parental education (as a marker of the family’s socioeconomic status), and family structure. In addition, to determine whether the results may have been affected by our choice of cut-off points for the victimisation variable, we also examined the relationship between victimisation and health by running the regression analysis using victimisation as a continuous variable in a sensitivity analysis. The results are presented in the form of odds ratios (OR) with 95% confidence intervals (CI). Following the lead of an earlier multi-country study that examined the effects of bullying on health among school-aged children [1] the analysis was stratified by sex. The analysis was conducted with Stata 12.0 (Stata Corp LP, College Station, Texas). Clustering within schools was adjusted for by using the clustered sandwich estimator.

## Results

Over 43% of the children had experienced occasional victimisation in the current school year with this figure being higher among boys (49.6%) than girls (38.7%) (Table 1). One-fifth (22.1%) of the children reported frequent victimisation. Again, this figure was much higher among boys (28.5%) than girls (17.6%). The prevalence of experiencing somatic symptoms had a wide range running from 10.3% of children reporting vomiting up to 54.6% of them having experienced headaches in the past 30 days. More girls reported experiencing symptoms in every outcome category with the sole exception of vomiting (boys 12.2% vs. girls 8.9%). Similar results were seen for the psychological symptoms. Just under one-quarter (24%) of girls had experienced symptoms of anxiety and depression whereas this figure was 15% for boys, while 33.5% of girls had experienced at least moderate levels of posttraumatic stress compared to 21.6% of boys.

**Table 1:** Prevalence of somatic and psychological symptoms, and peer victimisation among study respondents

	**Female% (95% CI)**	**Male% (95% CI)**	**Total% (95% CI)**
**Somatic symptoms**^¶^			
I had headaches	59.2 (56.2-62.2)	48.1 (45.8-50.4)	54.6 (52.4-56.8)
I had stomach aches	39.4 (37.0-41.8)	31.5 (28.5-34.5)	36.1 (34.2-37.9)
I had aches or pains	50.5 (47.5-53.4)	40.4 (35.9-44.9)	46.2 (43.2-49.2)
I had nausea	23.2 (20.3-26.1)	20.9 (18.1-23.7)	22.2 (20.3-24.1)
I felt sick	42.4 (39.3-45.4)	34.2 (30.5-37.9)	39.0 (35.9-42.0)
I had problems with my eyes	32.1 (28.9-35.3)	28.3 (24.2-32.4)	30.5 (27.3-33.8)
I had rashes or other skin problems	23.0 (20.0-26.0)	20.2 (18.2-22.3)	21.8 (20.1-23.5)
I was vomiting	8.9 (7.4-10.5)	12.2 (9.5-14.8)	10.3 (8.9-11.7)
**Psychological symptoms**^≠^			
Depression	24.3 (21.5-27.0)	15.1 (12.8-17.5)	20.5 (18.6-22.4)
Anxiety	24.6 (22.8-26.4)	15.2 (11.3-19.2)	20.8 (18.6-22.9)
Posttraumatic stress	33.5 (30.7-36.2)	21.6 (17.3-25.9)	28.5 (26.0-30.9)
**Peer victimisation**^#^			
Occasional	38.7 (36.4-41.1)	49.6 (46.6-52.5)	43.1 (41.3-45.0)
Frequent	17.6 (15.7-19.5)	28.5 (25.8-31.1)	22.1 (20.5-23.6)

^¶^Responses to somatic symptoms were dichotomised as not true and somewhat/certainly true.

^≠^Depression and anxiety symptoms were defined as the highest quintile of composite scores. Posttraumatic stress (PTS) relates to those with moderate or higher levels of PTS.

^#^Occasional and frequent school-based peer victimisation were based on 9 questions with answers: 0 (not at all), 1 (once), 2 (2–3 times), 3 (≥4 times). Those who answered 2–3 times or ≥4 times on at least one question were categorised as victims of occasional and frequent bullying respectively.

Peer victimisation at school was associated with increased odds for experiencing somatic health complaints with odds increasing as the severity of victimisation increased (Table 2). Compared with other girls who had not been victimised, those girls who were in the highest victimisation category were between 1.90 (problems with eyes) and 5.26 (aches and pains) times more likely to report somatic complaints with the corresponding figures for boys being 2.04 (headaches) and 4.36 (aches and pains – although higher odds (5.41) were seen for those boys with a score of 9–10 for this latter health outcome). Even the lowest level of victimisation (a score of 1–2) significantly increased the risk of experiencing many of the symptoms – and more than doubled the odds that girls would report having aches and pains (odds ratio (OR): 2.07; confidence interval (CI): 1.33-3.21).

**Table 2:** Association between peer victimisation and somatic symptoms

		**Female**		**Male**	
**Outcome**	**Victimisation**^**#**^	**%**	Adj. OR (95% CI)*	**%**	Adj.OR (95% CI)*
**Somatic symptoms**^**¶**^					
I had headaches	0	48.7	1.00	38.6	1.00
	1-2	56.9	1.44 (1.15-1.81)^b^	42.9	1.21 (0.83-1.75)
	3-5	61.3	1.81 (1.42-2.31)^c^	47.6	1.44 (1.04-1.99)^a^
	6-8	65.5	2.27 (1.65-3.11)^c^	54.0	1.80 (1.22-2.65)^b^
	9-10	67.2	2.33 (1.31-4.14)^b^	66.7	3.25 (2.38-4.43)^c^
	11-27	79.4	4.46 (2.51-7.94)^c^	57.1	2.04 (1.15-3.62)^a^
I had stomach aches	0	30.2	1.00	23.2	1.00
	1-2	36.5	1.34 (1.06-1.70)^a^	20.4	0.85 (0.59-1.23)
	3-5	43.2	1.85 (1.56-2.21)^c^	29.6	1.40 (0.94-2.08)
	6-8	44.4	2.00 (1.45-2.77)^c^	35.8	1.79 (1.31-2.45)^c^
	9-10	51.6	2.60 (1.71-3.95)^c^	39.7	2.28 (1.67-3.12)^c^
	11-27	53.3	2.82 (2.10-3.77)^c^	45.6	2.72 (1.90-3.90)^c^
I had aches or pains	0	33.1	1.00	24.8	1.00
	1-2	49.8	2.07 (1.33-3.21)^b^	33.1	1.46 (1.09-1.97)^a^
	3-5	54.3	2.71 (1.72-4.27)^c^	37.6	1.81 (1.28-2.57)^b^
	6-8	64.5	4.50 (3.16-6.41)^c^	43.7	2.21 (1.42-3.43)^c^
	9-10	64.1	4.09 (2.48-6.74)^c^	62.8	5.41 (3.75-7.79)^c^
	11-27	68.2	5.26 (3.25-8.52)^c^	58.8	4.36 (2.93-6.47)^c^
I had nausea	0	13.0	1.00	13.9	1.00
	1-2	22.0	1.95 (1.38-2.76)^c^	15.4	1.16 (0.69-1.97)
	3-5	23.8	2.24 (1.52-3.31)^c^	18.6	1.42 (0.87-2.31)
	6-8	32.0	3.46 (2.27-5.27)^c^	27.0	2.30 (1.11-4.77)^a^
	9-10	39.1	4.54 (2.12-9.72)^c^	29.5	2.60 (1.34-5.04)^b^
	11-27	33.6	3.74 (2.22-6.30)^c^	29.4	2.51 (1.53-4.11)^c^
I felt sick	0	28.3	1.00	21.6	1.00
	1-2	40.6	1.79 (1.41-2.27)^c^	29.4	1.52 (1.02-2.24)^a^
	3-5	45.5	2.28 (1.94-2.69)^c^	34.0	1.90 (1.43-2.53)^c^
	6-8	51.2	3.05 (2.11-4.40)^c^	39.2	2.26 (1.54-3.33)^c^
	9-10	62.5	4.63 (3.35-6.40)^c^	39.7	2.50 (1.61-3.87)^c^
	11-27	61.7	4.59 (2.59-8.15)^c^	49.4	3.43 (2.24-5.27)^c^
I had problems	0	27.5	1.00	17.9	1.00
with my eyes	1-2	29.8	1.12 (0.76-1.64)	17.1	0.96 (0.58-1.59)
	3-5	31.3	1.24 (0.81-1.89)	28.5	1.82 (1.28-2.59)^b^
	6-8	37.4	1.68 (1.30-2.16)^c^	36.5	2.62 (1.61-4.27)^c^
	9-10	45.3	2.25 (1.57-3.24)^c^	43.6	3.56 (2.12-5.96)^c^
	11-27	40.6	1.90 (1.15-3.14)^a^	37.5	2.79 (1.76-4.43)^c^
I had rashes or	0	14.1	1.00	10.9	1.00
other skin problems	1-2	18.0	1.36 (0.89-2.07)	16.3	1.59 (1.09-2.32)^a^
	3-5	23.4	1.83 (1.40-2.39)^c^	16.6	1.67 (0.77-3.65)
	6-8	34.3	3.21 (2.38-4.32)^c^	21.6	2.28 (1.34-3.89)^b^
	9-10	40.6	4.04 (2.59-6.31)^c^	35.9	4.65 (2.05-10.51)^c^
	11-27	37.4	3.69 (2.49-5.45)^c^	31.3	3.70 (2.38-5.74)^c^
I was vomiting	0	4.5	1.00	8.4	1.00
	1-2	7.1	1.66 (0.98-2.82)	9.1	1.12 (0.65-1.92)
	3-5	9.4	2.43 (1.38-4.28)^b^	9.5	1.18 (0.57-2.44)
	6-8	11.8	3.28 (1.41-7.62)^b^	7.9	0.96 (0.49-1.90)
	9-10	17.2	4.65 (1.69-12.79)^b^	20.5	2.91 (1.44-5.89)^b^
	11-27	14.0	4.10 (2.25-7.44)^c^	21.9	3.08 (1.89-5.02)^c^

^#^ School-based peer victimisation is a composite score based on 9 questions with answers: 0 (not at all) 1 (once) 2 (2–3 times) 3 (≥4 times).

*Adjusted for parental education, family structure and age.

^¶^ Responses to somatic symptoms were dichotomised as not true (reference) and somewhat/certainly true.

^a^P<0.05, ^b^P<0.01, ^c^P<0.001.

In terms of psychological symptoms, greater victimisation was also associated with higher odds for reporting worse mental health (Table 3). Compared to non-victims, girls and boys in the highest victimisation category were between 2.42 (girls) and 3.33 (boys) times more likely to have experienced anxiety, over 5 times more likely to report posttraumatic stress symptoms (girls OR: 6.45; CI: 5.00-8.32; boys OR: 5.09; CI: 3.31-7.82), and over 6 times more likely to have experienced symptoms of depression in the previous 30 days (girls OR: 6.09; CI: 3.18-11.66; boys OR: 6.63; CI: 4.91-8.95). When the victimisation variable was entered into the regression analysis as a continuous variable there was a significantly increased risk of experiencing all of the somatic and psychological health problems (p<0.001 for all health conditions (data not shown)).

**Table 3:** Association between peer victimisation and psychological symptoms

		**Female**		**Male**	
**Outcome**	**Victimisation**^**#**^	**%**	Adj. OR (95% CI)*	**%**	Adj. OR (95% CI)*
**Psychological symptoms**^**≠**^					
Depression	0	15.8	1.00	7.3	1.00
	1-2	20.2	1.42 (0.95-2.12)	10.5	1.46 (0.88-2.42)
	3-5	22.1	1.68 (1.14-2.48)^b^	14.2	2.22 (1.35-3.65)^b^
	6-8	37.6	3.95 (2.68-5.82)^c^	9.5	1.38 (0.88-2.17)
	9-10	36.7	3.55 (1.90-6.62)^c^	25.4	4.23 (2.25-7.94)^c^
	11-27	48.0	6.09 (3.18-11.66)^c^	32.9	6.63 (4.91-8.95)^c^
Anxiety	0	17.9	1.00	9.1	1.00
	1-2	21.0	1.23 (0.93-1.62)	13.6	1.65 (1.06-2.58)^a^
	3-5	26.6	1.70 (1.22-2.37)^b^	14.3	1.75 (1.07-2.86)^a^
	6-8	33.9	2.50 (1.65-3.78)^c^	13.5	1.62 (0.70-3.75)
	9-10	39.1	3.03 (1.83-5.02)^c^	21.1	2.57 (1.49-4.43)^b^
	11-27	34.0	2.42 (1.54-3.80)^c^	24.8	3.33 (1.90-5.85)^c^
Posttraumatic stress	0	22.9	1.00	13.9	1.00
	1-2	28.6	1.44 (1.12-1.86)^b^	12.4	0.96 (0.50-1.84)
	3-5	31.7	1.70 (1.14-2.54)^b^	17.3	1.42 (0.86-2.34)
	6-8	43.0	3.06 (2.15-4.35)^c^	24.1	2.09 (1.31-3.32)^b^
	9-10	61.1	6.15 (3.33-11.37)^c^	34.8	3.66 (1.85-7.26)^c^
	11-27	61.5	6.45 (5.00-8.32)^c^	45.3	5.09 (3.31-7.82)^c^

^#^ School-based peer victimisation is a composite score based on 9 questions with answers: 0 (not at all) 1 (once) 2 (2–3 times) 3 (≥4 times).

*Adjusted for parental education, family structure and age.

^≠^ Depression and anxiety symptoms were defined as the highest quintile of composite scores. Posttraumatic stress (PTS) refers to those with moderate or higher levels of PTS.

^a^P<0.05, ^b^P<0.01, ^c^P<0.001.

## Discussion

This study has shown that many adolescents experience peer victimisation in schools in northern Russia and that victimisation is strongly associated with psychological and somatic health problems. These findings are consistent with those of a recent meta-analysis of the consequences of bullying and victimisation for psychosomatic health [17]. Moreover, the relation we observed between experiencing more victimisation and having higher odds of poor health accords with findings from the earlier HBSC study conducted in 28 countries in Europe and North America [1] and a recent smaller-scale study from Norway [18] where a graded association was noted between the frequency of having been bullied and the likelihood of reporting different negative health outcomes. However, it was noticeable in the current study that in terms of somatic symptoms, for more than half of the symptoms there were higher odds among those girls and boys scoring 9–10. This was not observed for the psychological symptoms, where with the sole exception of anxiety among girls, those in the highest victimisation category (scoring 11–27) had the highest odds of reporting poor health. This and the fact that even relatively few instances of victimisation (i.e., scores of 1–2) were associated with poorer health outcomes in some cases highlights the necessity of future research using more finely graded categories of victimisation (i.e. relating to both type and intensity of victimisation) to better understand the effects of peer victimisation on adolescent health. Moreover, it seems unlikely that our findings are an artefact of the categorisation system we employed as when the victimisation variable was entered into the regression analysis as a continuous variable it was significantly associated with all of the health problems.

It has been suggested that stress may be the mechanism that links the experience of peer victimisation to negative health outcomes [19]. In relation to this, it is possible that social support, which can act to buffer the effects of stressful environments [20], may reduce the detrimental effects of peer victimisation on health outcomes [21]. This notion is supported by research that showed how differences in familial warmth protected against subsequent behavioural disorders in identical twins subject to victimisation [22] and by evidence that support from both parents and teachers may mitigate the effects of victimisation [23]. If support does act to mitigate the detrimental effects of victimisation on well-being this may explain the strong relationship we observed between victimisation and negative health outcomes in the current study. Specifically, some research indicates that the majority of Russian adolescents tend not to report experiencing peer victimisation and they feel that they cannot turn to teachers for help [8].

This suggests that the better training of teachers to recognise what have been described as the physical, psychosomatic and behavioural ‘warning signs’ of peer victimisation [24] may be one potentially effective intervention when it comes to addressing this issue. This could perhaps be one element in comprehensive school-based anti-bullying programmes which recent review articles have linked to a reduction in the occurrence of both bullying and victimisation in schools in other settings [25,26]. However, as other review evidence questions the extent to which school-based interventions reduce actual bullying behaviours [27], it is also important that possible actions to mitigate bullying and its effects are not restricted solely to schools. For example, other adults who come into contact with children – such as doctors – should also be made aware of the potential signs of bullying and what to do when children present with possible symptoms as a result of being bullied [28].

There are several possible limitations to this study that should be mentioned. First, as the data were self-reported with no means of verification there is the potential for reporting bias. Second, there is also a possibility of selection bias as we were only able to gather information from those children in school on the day of the survey. This may have been problematic as previous research has linked school absenteeism to victimisation [19]. Third, we equated frequency of victimisation with the intensity of the victimisation experience. However, the effects of being sworn at several times might differ markedly, say, from those of being badly physically beaten on only one occasion. Fourth, the questions on victimisation and health outcomes referred to different time periods i.e. this school year and the previous 30 days. The use of different reference periods may have introduced the possibility of bias into the study. Fifth, although we have followed previous authors in using 2–3 times as a cut-off to determine what constitutes victimisation, in the study we referenced, the precise definition was “‘2 or 3 times a month’ (in the past couple of months)” [15, p. 263]. In the current study however, the victimisation took place ‘During this school year’ i.e. the school year began in September and the survey was undertaken in March to May of the following year (more than 6 months after the beginning of the school year). Over this much longer time period the effects of experiencing 2–3 instances of victimisation might be very different from those suggested in the reference article. This indicates that the prevalence estimates from this study may not be strictly comparable with those from earlier studies using this victimisation cut-off point. Sixth, the somatic symptom ‘problems with eyes’ was not precisely defined and may have been interpreted in different ways by different respondents. Finally, the data we collected were cross-sectional so it is impossible to determine the order of events. A recent review of longitudinal research studies has suggested for example, that the relation between peer victimisation and internalising problems may be bi-directional where peer victimisation both leads to, and is a consequence of such problems [3].

## Conclusion

This study has shown that school-based peer victimisation is commonplace among adolescents in northern Russia and is associated with a variety of poorer health outcomes. In such circumstances a renewed focus needs to be placed on this issue by national, regional and school authorities. To achieve this more research from other parts of Russia will be necessary as this phenomenon is still little researched or understood, despite the strong negative impact it seems to be currently having on the health of Russian adolescents.

## Competing interests

The authors declare that they have no competing interests.

## Authors’ contributions

AS conceived the study idea and wrote the main body of the manuscript. AK analysed the data and helped draft and revise the manuscript. RK and VR designed and carried out the survey and commented on and helped revise the manuscript. MM and BR commented on and helped revise the manuscript. All authors have seen and approved the final version of the manuscript.

## Supplementary Material

Additional file 1 Peer Victimisation Scale. Click here for file
