# Supplementary material for: Peer victimisation and its association with psychological and somatic health problems among adolescents in northern Russia
Source: Child Adolesc Psychiatry Ment Health. 2013 May 14;7:15. doi: 10.1186/1753-2000-7-15 (PMC3661367; doi:10.1186/1753-2000-7-15)
Supplement: Additional file 1 — Peer Victimisation Scale. [file 1753-2000-7-15-S1.pdf]

# Peer Victimisation Scale

**These next questions ask you about bullying.**

| During this school year other kids **in school…** | Not at All | Once | 2-3 Times | 4 or More Times |
|---|---|---|---|---|
| **a.** called me names or swore at me. | **0** | **1** | **2** | **3** |
| **b.** tried to get me into trouble with my friends. | **0** | **1** | **2** | **3** |
| **c.** took something without permission or stole things from me. | **0** | **1** | **2** | **3** |
| **d.** made fun of me for some reason. | **0** | **1** | **2** | **3** |
| **e.** made me uncomfortable by standing too close or touching me. | **0** | **1** | **2** | **3** |
| **f.** punched, kicked or beat me up. | **0** | **1** | **2** | **3** |
| **g.** hurt me physically in some way. | **0** | **1** | **2** | **3** |
| **h.** tried to break or damage something of mine. | **0** | **1** | **2** | **3** |
| **i.** refused to talk to me or made other people not talk to me. | **0** | **1** | **2** | **3** |
